# A Co-Induction Technique Utilizing 4% Sevoflurane Followed by 0.75 mg/kg Propofol in Elderly Patients Undergoing Minimally Invasive Procedures: A Prospective Randomized Control Study

**DOI:** 10.3390/medicina56120682

**Published:** 2020-12-10

**Authors:** Omar A. Ababneh, Aiman M. Suleiman, Isam K. Bsisu, Subhi M. Al-Ghanem, Walid K. Samarah, Khaled R. Al-Zaben, Ibraheem Y. Qudaisat, Lubna A. Khreesha, Ghazi M. Al Edwan, Mujalli M. Murshidi

**Affiliations:** 1 Department of Anesthesia and Intensive Care, School of Medicine, The University of Jordan, Amman 11942, Jordan; alghanem@ju.edu.jo (S.M.A.-G.); walidzuabi@yahoo.com (W.K.S.); kalzaben@ju.edu.jo (K.R.A.-Z.); qudaisat@ju.edu.jo (I.Y.Q.); 2 Anesthesia and Intensive Care Department, Alabdali Clemenceau Hospital, Amman 11190, Jordan; Aiman.majed@yahoo.com; 3 Department of Otolaryngology, School of Medicine, The University of Jordan, Amman 11942, Jordan; l.khreesha@ju.edu.jo; 4 Department of Urology, School of Medicine, The University of Jordan, Amman 11942, Jordan; g.Aledwan@ju.edu.jo (G.M.A.E.); mujalli@ju.edu.jo (M.M.M.)

**Keywords:** sevoflurane, propofol, co-induction, geriatric anesthesia

## Abstract

*Background and Objectives:* Elderly patients constitute a large segment of healthcare receivers. Considering the functional deterioration of multiple organ systems with aging, achieving a safe perioperative approach is challenging. Our aim is to study the safety and effectiveness of a genuinely regimented co-induction technique in order to minimize anesthesia-related complications. *Materials and Methods:* One hundred and five patients were assigned to three groups according to the induction technique: propofol, sevoflurane and co-induction group. Inclusion criteria: patients with age ≥65 and American Society of Anesthesiologists physical status classification (ASA) II-III who underwent endoscopic urological procedures. The propofol group received a dose of 1.5 mg kg^−1^ of propofol over two minutes for induction. The sevoflurane group received 8% of sevoflurane and 100% oxygen through a plastic facemask with the fresh gas flow set at 8 L min^−1^. The co-induction group received 4% sevoflurane through plastic facemask for two minutes, followed by a 0.75 mg kg^−1^ dose of propofol. After ensuring full range jaw relaxation, the laryngeal mask airway (LMA) was inserted. *Results:* Overall, the co-induction technique had a favorable profile in terms of respiratory adverse events, while the sevoflurane group had a favorable profile in terms of hemodynamic stability. Furthermore, 24 (68.6%) patients receiving inhalational sevoflurane had episodes of transient apnea, which constitutes 77.4% of the 31 episodes of transient apnea in the studied sample (*p* < 0.001). Moreover, six (17.1%) patients in the sevoflurane group had an episode of partial laryngospasm (*p* = 0.034). Compared with the co-induction group, we found that the propofol group had significantly less systolic and diastolic blood pressures in the second minute, with *p* values of (0.018) and (0.015), respectively. *Conclusions:* The co-induction technique utilizing 4% sevoflurane at 8 L min^−1^ flow of oxygen inhaled over two minutes followed by 0.75 mg kg^−1^ of propofol achieved less respiratory adverse events compared with the sevoflurane group, and less hemodynamic instability compared with the propofol group.

## 1. Introduction

The co-induction technique refers to the employment of a combination of drugs to achieve a greater effect than each drug alone [1]. It is an approach that can greatly benefit elderly patients and patients with chronic diseases involving major organs in whom anesthesia could confer a greater risk if hemodynamics are altered. Many rationales stand behind the development of co-induction techniques. The most important one is the more balanced ratio of desired versus adverse effects [1]. When anesthetizing a patient, the anesthesiologist decides the proper induction technique based on several factors related to the patient and the planned procedure [2,3].

Propofol is a widely-used intravenous agent during induction and maintenance of general anesthesia [4,5,6]. Compared with other intravenous anesthetics, propofol provides optimal suppression of airway reflexes during laryngeal mask insertion with rapid onset of action and smooth recovery and has a relatively stable hemodynamic profile if dosed and administered properly [7,8,9,10]. However, its use is associated with some disadvantages such as bradycardia and hypotension on induction, depression of ventilation, and pain on injection [11,12,13]. Inhalational induction benefit to risk ratio has been studied extensively for non-pungent agents such as halothane and sevoflurane [14]. Considering its pharmacokinetic properties and absence of major side effects, sevoflurane represents a safe and reliable inhalational induction agent in various clinical settings [15]. Nevertheless, several adverse events were reported following its use such as postoperative nausea and vomiting [14]. Co-induction techniques employing inhalational and intravenous anesthetic agents consist of wide varieties and possibilities [16,17].

Elderly patients constitute a large segment of the general population receiving healthcare services worldwide. Taking into consideration the functional deterioration in several organ systems with aging, along with higher perioperative morbidity and mortality, this puts the anesthesiologist under the challenge of keeping the patient safe before, during, and after the surgery [18,19,20]. Hence, it is prudent to ask the question: how can we conduct anesthesia in a safe and efficient manner in these groups? As research on optimizing anesthetic approaches to these patients takes the lead, co-induction techniques should be investigated while taking recent proper anesthetic data and reviews into consideration. Our aim is to study the safety of a genuinely regimented co-induction technique in terms of efficiency of laryngeal mask airway (LMA) insertion, hemodynamic stability based on systolic blood pressure readings, and peri-induction adverse events, in order to minimize anesthesia-related complications.

## 2. Materials and Methods

### 2.1. Study Design

This prospective randomized control study was conducted at Jordan University Hospital, a tertiary hospital in Amman, Jordan. The study design and technique were approved by the institutional review board (IRB) committee at Jordan University Hospital (reference number: 10/2016/4597 and the clinical trial number: NCT04284644). Clinical trial registration was completed retrospectively on 24 February 2020 [21]. Moreover, the study was reported in accordance with the Consolidated Standards of Reporting Trials (CONSORT) statement (http://www.consort-statement.org/). The structure of this study is a comparative analysis of three distinct approaches to induction, each approach utilizes one of the following techniques: propofol alone, sevoflurane alone, or co-induction technique employing both drugs. It enrolled 105 patients who were assigned by a computer-generated randomization via Research Randomizer (www.randomizer.org) to three groups of equal numbers according to the induction technique used: propofol group, sevoflurane group and co-induction group. All groups received pre-oxygenation of three minutes duration at 8 L min^−1^ flow of oxygen. The propofol group received a dose of 1.5 mg kg^−1^ of propofol slowly over two minutes for induction. The sevoflurane group received 8% sevoflurane through sealed plastic facemask at 8 L min^−1^ flow of oxygen. The co-induction group received 4% sevoflurane through sealed plastic facemask at 8 L min^−1^ flow of oxygen for two minutes, followed by a dose of 0.75 mg kg^−1^ of propofol given slowly. After ensuring full range jaw relaxation, the laryngeal mask airway (LMA) was inserted.

### 2.2. Sampling

Informed written consents were obtained from all enrolled patients. Our inclusion criteria included patients with age ≥65 and American Society of Anesthesiologists physical status classification (ASA) II-III who underwent minimally invasive endoscopic urological procedures, such as cystoscopy, ureteroscopy, double-J catheter insertion, and transurethral bladder resection. The exclusion criteria contained patient refusal to any of the techniques after operating room admission, family history of malignant hyperthermia, prolonged surgeries that needed intubation due to unexpected events intraoperatively and body mass index (BMI) more than 35 kg m^−2^.

### 2.3. Materials

All patients were appropriately prepared by perioperative assessment, followed by 6 h fasting, during which they were kept on maintenance intravenous Ringer’s lactate solution. Patients did not receive any sedative drugs pre-operatively. In the operating room, patients in all groups were monitored according to American Society of Anesthesiologists (ASA) guidelines for minimally invasive procedures. Intravenous access with a 20 gauge cannula was established before induction. Preoxygenation for three minutes under 8 L min^−1^ flow of oxygen was performed. Patients were asked to hold a sponge ball in their dominant hands. Then, 1.5 mcg kg^−1^ of fentanyl was given intravenously. Two minutes following the fentanyl dose, every patient received the induction technique according to his pre-assigned group. In inhalational and co-induction groups, we used gas analyzer expiratory readings of sevoflurane to ensure adequate depth of anesthesia before LMA insertion.

Following the beginning of induction, we waited for sponge ball dropping from the dominant hand. After the drop, loss of verbal contact and eyelash reflex were checked, followed by checking jaw relaxation every 10 s for full range mobility, which indicates suitable conditions for LMA insertion [22]. We used classic LMA for this study. Appropriate size was primarily determined according to patient’s weight and was replaced in patients who had more than two trials by a one size larger version. Insertion was completed utilizing a two person technique. After insertion, placement was confirmed by adequate manual ventilation, chest rising, normally shaped capnography and fiberoptic laryngoscopy. All respiratory adverse events were assessed throughout until successful placement of LMA. We included vital signs’ records until the start of the surgical procedure or up to 15 min after induction of anesthesia, whichever comes first, to investigate the anesthetics’ effect on hemodynamics without the interference of surgical factors. For treatment of complications, all complications were approached and treated accordingly. For instance, transient apnea was managed by assisting ventilation manually, once apnea is noted via face mask. Laryngospasm was mainly managed by applying 100% oxygen, clearing the oropharynx, and the deepening of anesthesia. Hypotension was managed by head-down positioning, applying 100% oxygen, and repeating the blood pressure reading immediately, and in case the reading was still hypotensive, ephedrine was given accordingly, and the total ephedrine dose was recorded. Anesthesia afterwards was maintained using sevoflurane inhalation one minimum alveolar concentration (MAC) end-tidal sevoflurane throughout the surgery for the three groups. Ventilatory settings were maintained at appropriate respiratory rates and tidal volumes according to end-tidal CO_2_ readings, and patients were put on spontaneous ventilation once full respiratory capacity was restored.

The primary target of this study was to evaluate the relative safety of our co-induction regimen in terms of efficiency of LMA insertion, hemodynamic stability based on systolic blood pressure readings, and peri-induction adverse events. We assessed time to LMA insertion, number of trials for LMA insertion, succession of LMA insertion, complications related to patient’s airway and respiration (cough, apnea, laryngospasm), hemodynamic alterations and satisfaction regarding the technique used. Of particular, we used Brimacombe score, which is a fiberoptic scoring system that assesses the best placement of laryngeal mask according to the view [23]. For respiratory complications, we considered the onset of audible inspiratory wheezes “stridor” that were abolished upon applying minimal positive expiratory pressure (partial laryngospasm), and sudden inspiration at mid-expiration followed by a pause in respiration of ≥5 s (transient apnea) [24]. For patient’s satisfaction, we used a modified version of a multivariable ordinal scale that covers many aspects related to induction and was used in Moran et al.’s 2007 study [24], in which we investigated the comfortability of the mask sealing, the smell of the mask and volatile anesthetics, their satisfaction with the induction technique, and whether they would like to use the same method should they need any future interventions.

### 2.4. Statistical Analysis

Based on the effect size calculated by Moran et al. [25], who stated that 10% difference in the percent change of the systolic blood pressure (SBP) relative to its baseline is considered statistically significant. With assumption of 80% test power and a type I error of 0.05, the required sample in each arm was 16. Our study included 35 patients in each arm to increase the power of the study further.

We used Statistical Package for the Social Sciences (SPSS) version 21.0 (Chicago, IL, USA) for analyzing the collected data. Age, BMI, time to weight drop, time for LMA insertion, SBP, diastolic blood pressure (DBP), mean arterial pressure (MAP), heart rate (HR) and oxygen saturation pressure (SpO_2_) were first analyzed using the Kolmogorov–Smirnov test for normality, followed by one-way analysis of variance (ANOVA) whenever data were normally distributed, and the Kruskal–Wallis test by ranks for non-normally distributed variables. When statistically significant, one-way ANOVA was followed by the least significance difference (LSD) post hoc analysis. The Mann–Whitney U test was used to compare sevoflurane levels at different timings between inhalational sevoflurane and co-induction groups. The chi squared test was used to compare the three groups in terms of gender, ASA score, airway assessment, number of trials of LMA insertion, episodes of transient apnea, cough, laryngeal spasm, gagging, increased salivation, limb movement, the use of ephedrine, and patients’ satisfaction. A *p* value less than 0.05 was considered as a threshold for statistically significant correlations.

## 3. Results

Overall, 105 patients were enrolled in this study, with 35 patients being assigned to each of the three groups. None of the patients enrolled met our exclusion criteria. The mean age of the study sample was 71.1 (4.7) years, of which 82 (78.1%) were males and 23 (21.9%) were females. The demographic data and airway assessment of the patients are presented in Table 1. The comparison between the three groups in terms of demographics data and airway assessment did not show any significant difference between the three studied groups.

Upon investigating the time to sponge ball drop, the one-way ANOVA test demonstrated a significant difference in the mean duration between the three groups (*p* < 0.001). The post hoc comparison of LSD showed that the propofol group needed significantly less time when compared with the other two groups (*p* < 0.001). In addition, the inhalational sevoflurane group needed significantly less time to weight drop, when compared with the co-induction group (*p* = 0.021).

Interestingly, 24 (68.6%) patients receiving inhalational sevoflurane had episodes of transient apnea, which constitutes 77.4% of the 31 episodes of transient apnea in the studied sample (*p* < 0.001). Moreover, six (17.1%) patients receiving inhalational sevoflurane had an episode of partial laryngospasm (*p* = 0.034) (Table 2).

**Figure 1:**
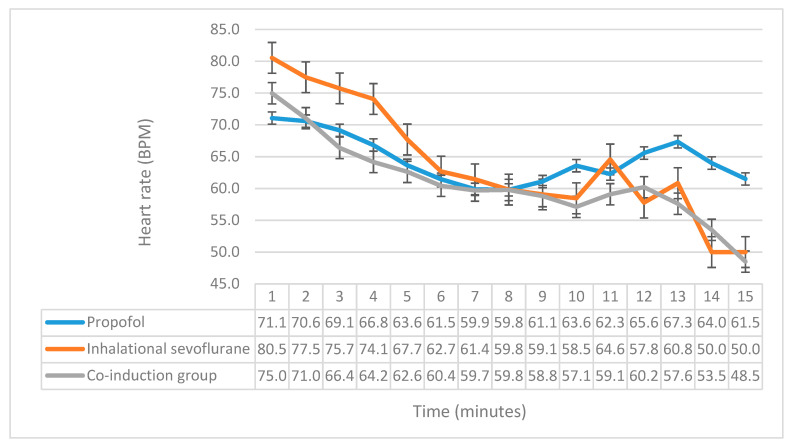
A comparison of the mean heart rate between the three groups. The *x*-axis represents the time in minutes, while the *y*-axis represents the heart rate in beats per minute, and the y-error bars represent standard error.

In Figure 1, we demonstrated the comparison between the three groups in their HR after induction of anesthesia. No significant difference was found between the three groups in terms of the HR during and after the induction (*p* > 0.05), except at the fourth minute, where *p* = 0.047.

**Figure 2:**
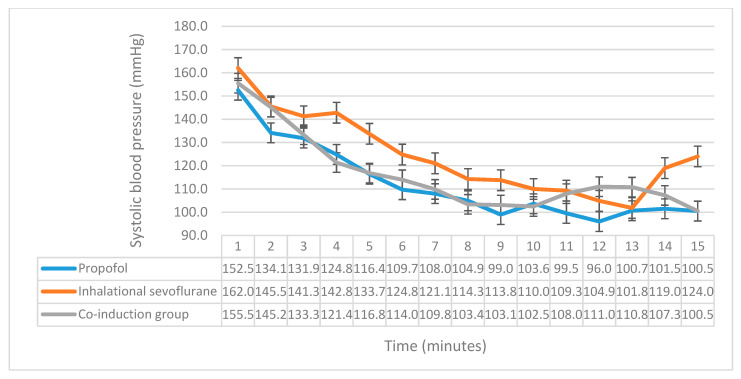
A comparison of the systolic blood pressure between the three groups. The *x*-axis represents the time in minutes, while the *y*-axis represents the systolic blood pressure, and the y-error bars represent standard error.

**Figure 3:**
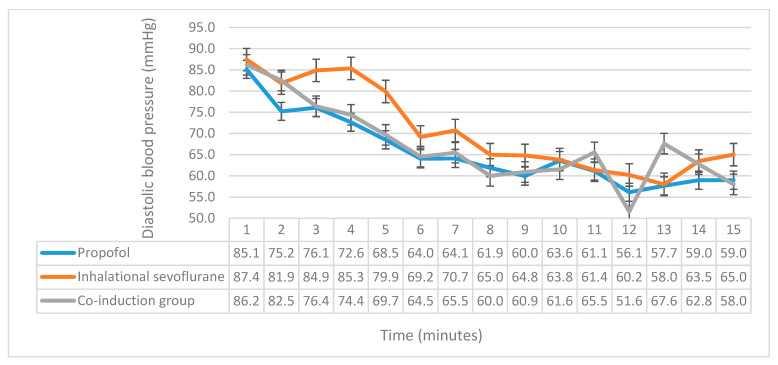
A comparison of the diastolic blood pressure between the three groups. The *x*-axis represents the time in minutes, while the *y*-axis represents the diastolic blood pressure, and the y-error bars represent standard error.

**Figure 4:**
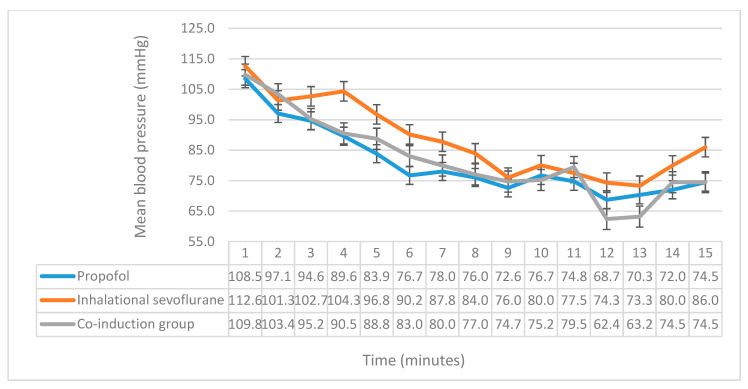
A comparison of the mean arterial pressure between the three groups. The *x*-axis represents the time in minutes, while the *y*-axis represents the mean arterial blood pressure, and the y-error bars represent standard error.

SBP, DBP and MAP monitoring revealed significant differences between the three groups. Significant differences were found in SBP in the second (*p* = 0.01), fourth (*p* = 0.009), fifth (*p* = 0.007), sixth (*p* = 0.019), and ninth (*p* = 0.015) minutes (Figure 2). Post hoc comparison of LSD showed that the propofol group had significantly lower SBP in the second (*p* = 0.003), fourth (*p* = 0.005), fifth (*p* = 0.002), sixth (*p* = 0.006), and ninth minutes (*p* = 0.008) when compared with the inhalational sevoflurane induction group, while when compared with the co-induction group, we found that the propofol group only had significantly less SBP in the second minute (*p* = 0.018). Upon comparing SBP between the co-induction group and the inhalational group, a significant difference was found in the fourth (*p* = 0.013), fifth (*p* = 0.038), and ninth (*p* = 0.026) minutes. The comparison of DBP yielded a significant difference in the second (*p* = 0.018), third (*p* = 0.02), fourth (*p* < 0.001), and fifth (*p* = 0.001) minutes (Figure 3). The propofol group had significantly less DBP when compared with the inhalational sevoflurane induction group in the second (*p* = 0.013), third (*p* = 0.011), fourth (*p* < 0.001), and fifth (*p* < 0.001) minutes. Upon comparing between the propofol and co-induction groups, DBP was significantly less in the second (*p* = 0.015) minute for propofol. The co-induction group had significantly less DBP than the sevoflurane induction group in the third (*p* = 0.023), fourth (*p* = 0.001), and fifth (*p* = 0.014) minutes. Moreover, we investigated the differences in MAP, and found significant differences in the fourth (*p* = 0.001), fifth (*p* = 0.004), and sixth (*p* = 0.010) minutes (Figure 4). The propofol group had significantly less MAP than the sevoflurane induction group in the fourth (*p* = 0.001), fifth (*p* = 0.001), and sixth (*p* = 0.002) minutes. Additionally, the co-induction group had less MAP when compared with the sevoflurane induction group in the fourth (*p* = 0.002), and fifth (*p* = 0.044) minutes. Moreover, we investigated the differences in patients’ satisfaction between the three groups using the aforementioned multivariable ordinal scale, with no significant differences being found between them (Table 3).

## 4. Discussion

Co-induction relies on the principle of decreasing doses of the administered drugs when combining them, hence benefiting from their synergism, and decreasing their side effects [1]. It also provides a more balanced outcome at lower costs. In addition, decreasing the dosage of drugs is beneficial in decreasing wastage and pollution in inhalational induction [26].

In our study, we found that the co-induction technique had a favorable profile in terms of respiratory adverse events when compared with the inhalational group, but more hemodynamic instability was noted. In comparison with the propofol group, the co-induction technique had a better hemodynamic stability.

In terms of hemodynamic stability and reliability of pharmacokinetics and pharmacodynamics, we found sevoflurane to be the best agent to be used as it reasonably maintains cardiac output and does not have a pungent smell [27]. Propofol is one of the most widely-used intravenous agents for induction. It has a relatively safe profile and reliable kinetics, and provides an optimal suppression of airway reflexes, which makes it a tempting agent for co-induction [7].

Many studies have evaluated the efficiency of co-induction techniques in elderly and risky patients. Of those with a relevant safety approach, a study conducted in Worthing Hospital, UK, in 2008, allocated 38 patients into two groups, a co-induction group who received 0.75 mg kg^−1^ propofol followed by 8% sevoflurane, and an inhalational group who received 8% sevoflurane alone [25]. Starting the induction with propofol might hinder the ventilation to variable degrees, especially in the elderly, which in turn decreases the benefits of sevoflurane as a second agent in the co-induction technique. In addition, opening sevoflurane at 8% violates the concept of co-induction, which is to decrease the dosages of the drugs and still achieve the same pharmacological effects. Finally, opening sevoflurane at 8% while ventilation is hindered will maximize the volume of pollution through a semi-open plastic facemask used for inhalational induction, and probably add to the possible risks of anesthesiologists’ exposure to inhalational agents.

Based on the aforementioned scope, we developed a technique that utilizes a lower concentration of sevoflurane (4%) followed by a reduced dose of propofol (0.75 mg kg^−1^). This is undertaken to benefit from the patient’s maximum ability to ventilate at the beginning of induction while achieving adequate suppression of airway reflexes when propofol is given before the insertion of LMA. All patients enrolled in our study had similar characteristics in terms of age, gender distribution, weight, height, ASA score, and the prevalence of chronic medical diseases. No significant difference was noted in upper airway assessment between the three groups, and all groups achieved an optimal Brimacombe score of four in approximate ratios.

Concerning time to ball drop from the dominant hand, which was allocated by the team of the study as a sign to check full jaw relaxation for suitability of LMA placement, it was prolonged in inhalational and co-induction techniques. This can be attributed to the faster onset of propofol compared with the slow build-up of sevoflurane inside the lungs [28]. Average time required for sevoflurane induction is dramatically increased with age [29]; hence, this finding can be attributed to the age of the chosen sample. Considering the safety provided by sevoflurane in terms of organ protection and postoperative complications, the importance of delay time is relatively minor [14]. Nevertheless, no significant difference was shown between the three groups in terms of time to LMA insertion and time to start of surgery. Number of trials and time for successful LMA insertion showed comparable results with no significant difference between all groups. This highlights the practicality of the suggested alternative co-induction technique.

Coughing can be initiated through pulmonary irritant receptors in response to gases entering airways [30]. Although noted more in inhalational group, the difference is not significant. Propofol is reported to induce violent coughing at induction doses of ≥2 mg kg^−1^, but this effect is more prominent in toddlers [31].

Respiratory complications constitute a major risk factor for morbidity and mortality in the postoperative period [32]. The prevalence of respiratory diseases is higher among the elderly population [33]. Respiratory adverse events showed significant difference between the three groups; in particular, transient apneas and partial laryngospasms were significantly higher in the inhalational group. Sevoflurane causes more respiratory depression than halothane [34], and transient apnea is a known complication of sevoflurane induction. Interestingly, 24 (68.6%) patients receiving inhalational sevoflurane had episodes of transient apnea. Nevertheless, sevoflurane-induced apneas are mainly related to the mode of administration [18]. A lower concentration dial with incremental mode of administration (slowly approaching the desired value) can decrease the frequency of transient apneas [35]. Transient apnea can be the first sign of more ominous events such as aspiration, laryngospasm, and negative pressure pulmonary edema. In regard to laryngospasm, a partial laryngospasm manifests as stridor or inefficient thoracic movements [36]. It can progress to full laryngospasm with complete airway obstruction within a matter of seconds [37]. It occurs due to periglottic triggers that can be mechanical, chemical, or thermal [38]. Although sevoflurane is a non-pungent agent and one of the least irritant inhalational agents, it can trigger laryngospasm at high concentrations in elderly patients [38]. Anesthesiologists should try to balance the risk of laryngospasm related to sudden delivery of large concentrations of sevoflurane with the risk of laryngospasm due to inadequate depth of anesthesia if very small doses were delivered.

In regard to hemodynamic alterations, the co-induction technique achieved optimal paradigms in diastolic and systolic blood pressures when compared with the propofol group, despite no significant difference in MAP. When compared with the co-induction group, we found that the propofol group only had significantly less SBP on the second minute (*p* = 0.018). The importance of maintaining systolic and diastolic blood pressure relies on sustaining enough coronary blood supply as blood flows to the heart and major organs [39]. Recently, there is growing evidence that shows that even brief duration of low SBP or MAP can be harmful [40]. Adverse events such as myocardial infarction (MI), acute renal failure and cardiac failure were all reported following any duration of hypotension that requires intervention [41,42]. In addition, intraoperative hypotension is one of the most encountered risk factors associated with death in relation to anesthesia [43]. Elderly patients are more susceptible to complications; hence, maintaining any of the vital parameters is crucial. In all the patients in our study, MAP was maintained ≥65 mmHg. In terms of ephedrine usage, no significant difference was found between the three groups.

Finally, there were no significant difference in patient’s satisfaction between the three groups; hence, using the technique that confers the best outcomes in other parameters is recommended.

The main limitation of this study was that this study was powered only for systolic blood pressure measurement. Other important limitations include the logistic lack of targeted control infusion-based administration of propofol and bispectral index (BIS) value.

We recommend further studies to evaluate the difference between co-induction and sevoflurane induction in this group. Moreover, with the limited literature available on the effectiveness of the co-induction technique in reducing respiratory adverse events when compared to inhalational induction, future studies powered to primarily investigate this outcome are highly encouraged. We realize our study was exclusive to minimally invasive urological procedures, where effects of surgical interventions and the duration of anesthesia might affect perioperative hemodynamics and postoperative patients’ satisfaction.

## 5. Conclusions

In conclusion, the co-induction technique utilizing 4% sevoflurane at 8 L min^−1^ flow of oxygen inhaled over two minutes followed by 0.75 mg kg^−1^ of propofol achieved less respiratory adverse events compared with the sevoflurane group, and less hemodynamic instability compared with the propofol group. Considering the major negative consequences related to these adverse events and alterations, we recommend this technique as a safe approach in elderly patients.

## Figures and Tables

**Table 1:** A comparison between the three groups in terms of patients’ demographics and airway assessment.

		Group			Total	*p*-Value
		Propofol (*n* = 35)	Inhalational Sevoflurane (*n* = 35)	Co-Induction Group (*n* = 35)		
Age(year)	Median (IQR)	70 (68–73)	71 (68–76)	70 (66–73)	70 (68–75)	0.306
Weight (kg)	Median (IQR)	75 (70–87)	82 (71–90)	79 (70–90)	80 (70–90)	0.789
Height (m)	Median (IQR)	1.7 (1.60–1.75)	1.7 (1.65–1.77)	1.7 (1.67–1.77)	1.7 (1.65–1.77)	0.863
BMI (kg m^−2^)	Median (IQR)	28.1 (24.4–29.6)	27.7 (24.8–32)	27.3 (23.7–30.5)	27.7 (24.3–30.5)	0.789
Gender	Males	27 (77.1)	26 (74.3)	29 (82.9)	82 (78.1)	0.677
	Females	8 (22.9)	9 (25.7)	6 (17.1)	23 (21.9)	
ASA score	II	20 (57.1)	15 (42.9)	13 (37.1)	48 (45.7)	0.224
	III	15 (42.9)	20 (57.1)	22 (62.9)	57 (54.3)	
Airway examination						
Mouth opening (cm)	>3	35 (100)	34 (97.1)	35 (100)	104 (99.0)	0.364
Mallampati score	1.00	6 (17.1)	9 (25.7)	9 (25.7)	24 (22.9)	0.219
	2.00	22 (62.9)	19 (54.3)	13 (37.1)	54 (51.4)	
	3.00	7 (20.0)	7 (20.0)	13 (37.1)	27 (25.7)	
TM distance (cm)	>6	34 (97.1)	34 (97.1)	34 (97.1)	102 (97.1)	0.558
head and neck movement	NL	35 (100)	33 (94.3)	35 (100)	103 (98.1)	0.13
Trachea	Central	34 (97.1)	35 (100)	35 (100)	104 (99.0)	0.364
FOL View	1	2 (5.7)	3 (8.6)	2 (5.7)	7 (6.7)	0.645
	2	5 (14.3)	7 (20.0)	4 (11.4)	16 (15.2)	
	3	7 (20.0)	3 (8.6)	3 (8.6)	13 (12.4)	
	4	21 (60.0)	22 (62.9)	26 (74.3)	69 (65.7)	

IQR: interquartile range; BMI: body mass index (kg/m2); ASA: American Society of Anesthesiologists; FOL: fiberoptic laryngoscopy; TM distance: thyromental distance; cm: centimeter. Data are presented in *n* (%) and median, IQR.

**Table 2:** A comparison between the three groups in terms of intraoperative events, adverse events, and follow-up.

		Group			Total	*p*-Value
		Propofol (*n* = 35)	Inhalational Sevoflurane (*n* = 35)	Co-Induction Group (*n* = 35)		
Time to ball drop (sec)	Median (IQR)	80 (60–104)	120 (95–135)	150 (125–165)	120 (85–150)	<0.001
LMA size	3	3 (8.6)	4 (11.4)	5 (14.3)	12 (11.4)	0.363
	4	20 (57.1)	12 (34.3)	14 (40.0)	46 (43.8)	
	5	12 (34.3)	19 (54.3)	16 (45.7)	47 (44.8)	
Number of trials for LMA insertion	1	29 (82.9)	24 (68.6)	29 (82.9)	82 (78.1)	0.248
	2	4 (11.4)	10 (28.6)	6 (17.1)	20 (19.0)	
	3	2 (5.7)	1 (2.9)	0 (0)	3 (2.9)	
Time for LMA insertion after ball drop (sec)	Median (IQR)	6 (5–10)	10 (5–20)	9 (5–10)	8 (5–10)	0.313
Time to surgery from preoxygenation (min)	Median (IQR)	10 (8–11)	11 (9–12)	10 (9–11)	10 (8–11)	0.468
Respiratory adverse events		4 (11.4)	26 (74.3)	4 (11.4)	34 (32.4)	<0.001
	Cough	1 (2.9)	4 (11.4)	1 (2.9)	6 (5.7)	0.204
	Transient apnea	3 (8.6)	24 (68.6)	4 (11.4)	31 (29.5)	<0.001
	Partial laryngospasm	1 (2.9)	6 (17.1)	1 (2.9)	8 (7.6)	0.034
Limb movement		14 (40.0)	15 (42.9)	11 (31.4)	40 (38.1)	0.592
Gagging		10 (28.6)	11 (31.4)	10 (28.6)	31 (29.5)	0.955
Increased salivation		1 (2.9)	2 (5.7)	2 (5.7)	5 (4.8)	0.811
Sevoflurane level	1st minute	-	4.4 ± 0.4	2.1 ± 0.3	3.2 ± 1.2	<0.001
	2nd minute	-	5.0 ± 0.8	2.7 ± 0.3	3.8 ± 1.3	<0.001
	3rd minute	-	5.3 ± 0.4	3.0 ± 0.4	4.1 ± 1.2	<0.001
	4th minute	-	5.4 ± 0.4	3.2 ± 0.2	4.3 ± 1.2	<0.001
Need of Ephedrine during induction		9 (25.7)	5 (14.3)	3 (8.6)	17 (16.2)	0.14
Mean dose of Ephedrine among cases for which it was administered		7.0 ± 6.2	8.40 ± 5.4	40 ± 1.7	0.265	

IQR: interquartile range; LMA: laryngeal mask airway; sec: seconds; min: minutes. Data are presented in n (%) and median, IQR.

**Table 3:** A comparison between the three groups in terms of patients’ satisfaction with the applied anesthetic method.

Characteristic		Group			Total	*p*-Value
		Propofol (*n* = 35)	Inhalational Sevoflurane (*n* = 35)	Co-Induction Group (*n* = 35)		
Do you feel uncomfortable with mask sealing?	No	32 (91.4)	31 (88.6)	34 (97.1)	97 (92.4)	0.388
	Yes	3 (8.6)	4 (11.4)	1 (2.9)	8 (7.6)	
How do you feel about the gas smell?	Pleasant	13 (37.1)	7 (20.0)	10 (28.6)	30 (28.6)	0.573
	Neutral	18 (51.4)	22 (62.9)	20 (57.1)	60 (57.1)	
	Unpleasant	4 (11.4)	6 (17.1)	4 (11.4)	14 (13.3)	
	Very unpleasant	0 (0)	0 (0.0)	1 (2.9)	1 (1.0)	
How do you feel about the start of induction?	Pleasant	22 (62.9)	20 (57.1)	22 (62.9)	64 (61.0)	0.451
	Neutral	12 (34.3)	12 (34.3)	13 (37.1)	37 (35.2)	
	Unpleasant	1 (2.9)	3 (8.6)	0 (0)	4 (3.8)	
Will you accept to have this method of induction again?	Definitely would like to	26 (74.3)	21 (60.0)	29 (82.9)	76 (72.4)	0.317
	Probably would like to	5 (14.3)	8 (22.9)	5 (14.3)	18 (17.1)	
	Not sure	3 (8.6)	5 (14.3)	0 (0.0)	8 (7.6)	
	Probably would not	1 (2.9)	1 (2.9)	1 (2.9)	3 (2.9)	

Data are presented in *n* (%) and median, IQR.
